# MiR-20a-5p regulates gemcitabine chemosensitivity by targeting RRM2 in pancreatic cancer cells and serves as a predictor for gemcitabine-based chemotherapy

**DOI:** 10.1042/BSR20181374

**Published:** 2019-05-07

**Authors:** Huimin Lu, Shan Lu, Dujiang Yang, Ling Zhang, Jun Ye, Mao Li, Weiming Hu

**Affiliations:** Department of Pancreatic Surgery, West China Hospital, Sichuan University, Chengdu City, Sichuan Province 610041, PR. China

**Keywords:** gemcitabine chemosensitivity, miR-20a-5p, pancreatic cancer, RRM2

## Abstract

Ribonucleotide reductase subunit M2 (*RRM2*) acts as an important gemcitabine resistance-related gene in pancreatic cancer (PC). Here, we aimed to investigate the potential microRNA that regulates gemcitabine chemosensitivity by targeting RRM2 and explores the clinical significance of candidate miRNA in PC. MTT assay and Western blot analysis revealed that long-time gemcitabine treatment in PC cells induced drug resistance and RRM2 increase, and silence of RRM2 blocked gemcitabine resistance. Among the predicted eight RRM2-related microRNAs, the expression of miR-20a-5p showed the most significant discrepancy between gemcitabine-resistant cells and parental cells. Furthermore, the Dual-Luciferase reporter gene assay indicated that miR-20a-5p directly targeted RRM2 3′UTR, thus inhibited expression of RRM2 and overcame gemcitabine resistance of PC cells. Retrospective study suggested that plasma miR-20a-5p level was correlated with gemcitabine resistance in PC patient. ROC curve showed that miR-20a-5p abundant level might predict gemcitabine resistance with an AUC of 89% (*P*<0.0001). Additionally, the PFS of patients with high and low expression levels miR-20a-5p was 2.8 and 4.5 months (*P*<0.001), respectively. Taken together, our results suggests that miR-20a-5p regulated gemcitabine chemosensitivity by targeting RRM2 in PC cells and could serve as a predictor for predicting the efficacy of gemcitabine-based chemotherapy in first-line treatment of PC patients.

## Introduction

Pancreatic cancer (PC) has the highest mortality rate of all major cancers, with an overall survival rate of only approximately 5%. The mortality rates of PC in China showed an approximately 1.14-fold increase, from 2.85/100,000 in 2003 to 3.26/100,000 in 2011, with an annual percentage change of 1.68 [1]. Chemotherapy is the essential adjunct treatment for PC, and gemcitabine is currently being utilized. But resistance to gemcitabine is a major obstacle to effective chemotherapy for PC. A better understanding of the molecular mechanism of PC resistance against gemcitabine is urgently needed.

Ribonucleotide reductase (RR) is an enzyme that catalyzes the formation of deoxyribonucleotides from ribonucleotides, the critical process of cell replication [2]. It is overexpressed in a number of solid tumors including pancreatic [3]. Evidence suggest that RR plays a positive role in tumor cell proliferation and metastasis as well as the development of resistance to nucleoside analogs used in PC chemotherapy, including gemcitabine [4]. RR consists of an α subunit encoded by *Rrm1* and a β subunit encoded by *Rrm2*. RRM1–RRM2 holoenzyme provides dNTPs for S-phase nDNA replication and repair in proliferating cells. High-level RRM1 expression correlates with poor responses to gemcitabine, which directly targets RRM1 [5]. Whereas low RRM2 expression in pancreatic cancers is predictive of greater response to gemcitabine [6]. Meanwhile, RRM2 knockdown also overcomes cisplatin resistance in cultured cells [7]. RRM2 expression has been shown to be induced in chemoresistant cells by gene amplification, transcriptional activation, and perhaps other unidentified mechanisms [8]

Accumulating data has showed that microRNAs (miRNAs) were involved in the pathogenesis of PC. MiRNAs are small, endogenous, noncoding RNA molecules comprising 18–24 nucleotides that cause degradation of mRNA or inhibit translation by binding with complementary sequences in the 3′-untranslated region (3′-UTR) of their target mRNAs [9]. miRNA regulation of gene expression plays an important role in tissue differentiation, cell proliferation, apoptosis and chemoresistance [10,11]. In addition, resistance to certain dNTP analogs is linked to differential miRNA expression [12]. However, the role of miRNA in sensitivity of PC cells to chemotherapy is not fully understood and needs to be further investigated.

Hence, to study the potential interplay between miRNA and RRM2 and to further explore the opportunity of utilizing miRNAs for pancreatic cancer therapeutics, we sought to determine the direct impact of eight RRM2-related miRNAs, predicted by TargetscanSites, picTarSites, RNA22Sites, PITASites or miRandaSites.org algorithms, on acquired gemcitabine resistance. The function of one dramatically decreased miRNA, miR-20a-5p, in gemcitabine resistance was focused on sequential study.

## Methods

### Cell culture

The human pancreatic cancer cell line MIA-PaCa2 and human embryonic kidney cell line 293 (HEK293) were obtained from the American Type Culture Collection (ATCC). Gemcitabine-resistant MIA-PaCa2 cells (MIA-PaCa2-GEM) were selected by continuous treatment of MIA-PaCa2 cells in increasing concentrations of gemcitabine when the confluence of cells reached 40∼60% resulting in subclones resistant to 200 nM gemcitabine as described [13]. All cell lines were cultured in DMEM (18 mmol/l glucose) supplemented with 10% FCS and 5% HEPES.

### Patient data, plasma and tissues

All patients in the present study were histologically diagnosed with pancreatic adenocarcinoma. The diagnoses were established by conventional clinical and histological criteria according to the World Health Organization (WHO). The research has been carried out in accordance with the World Medical Association Declaration of Helsinki, and that all subjects provided written informed consent. Patient plasma and Data were obtained under the approval of the ethical committee of West China Hospital. None of these patients underwent any chemotherapy or radiotherapy, and none had metachronous multiple cancers in other organs. Besides, patient lacks of clinical follow-up information for study purposes was excluded. Total 73 patients received the gemcitabine-based chemotherapy as first-line treatment, including gemcitabine plus cisplatin, gemcitabine plus Nab-paclitaxel or gemcitabine plus Oxaliplatin. The dosages were described previously [14,15]. PCa tissues were isolated by enzymatic digestion and gradient density centrifugation. Blood samples were collected before chemotherapy and then centrifuged at 3000 × *g* for 10 min. All samples were stored in RNase- and DNase-free tubes at –80°C before use. According to the progression-free-survival (PFS), we divided patients into two groups: PFS ≤ 3 months (GEM resistance, *n*=34) and PFS ≥ 3 months (GEM nonresistance, *n*=39). The two groups have no significant difference in patients’ sex, age, phase and chemotherapy regimens. On other hand, according to the expression of miR-20a-5p, patients were divided into high miR group (*n*=36, GEM resistance: 11, GEM nonresistance: 27) and low miR group (*n*=37, GEM resistance: 25, GEM nonresistance: 12).

### RNA exaction

Plasma RNA and cultured cells RNA isolation were respectively performed using a Blood Total RNA Isolation Kit (RP4001, BioTeke, Beijing, China) and Trizol reagent (Invitrogen, Carlsbad, CA, U.S.A.), according to manufacturer’s protocol.

### Reagents

Gemcitabine solution (Eli Lilly, Indianapolis, IN, U.S.A.) was diluted in cell culture medium to a 100-μM stock and then added directly to cell culture medium according to the purpose of the experiments. The final concentrations of the solvents in medium were 0.1% or less.

### Cell viability

Viability was measured using 3-(4,5-dimethylthiazol-2-yl)-2, 5-diphenyltetrazolium bromide (MTT) assay. MIA-PaCa2 or MIA-PaCa2-GEM Cells were plated in 96-well plates overnight before the gemcitabine or vehicle treatment. For siRNA or miR mimic/inhibitor treatment, the cells were transfected with RNA Oligoribonucleotides in the presence or absence of 100 nM gemcitabine. At the end of the treatment, 10% v/v of 5 mg/ml solution of MTT agent (Sigma-Aldrich) was added for 2 h. The medium was then removed and the cells were dissolved in DMSO (Sigma-Aldrich). Relative cytotoxicity was determined by measuring the absorbance at 570 nm using a luminometer (Molecular Devices, U.S.A.).

### Apoptosis

Cells were stained with FITC-conjugated Annexin V (BD Biosciences, Heidelberg, Germany) and propidium iodide (5 mg/ml) (Sigma-Aldrich) and analyzed by Flow Cytometer (Beckton Dickinson, BD Biosciences, Germany), as described [16].

### qRT-PCR

The RNA concentrations were measured with a NanoDrop 2000 Spectrophotometer (Nano Drop Technologies, Wilmington, U.S.A.) and 500 ng total RNA or miRNA were reverse transcribed to cDNA using the High Capacity RNA to cDNA KIT (Thermo Fisher Scientific GmbH, Dreieich, Germany) or the Taqman microRNA Reverse Transcription kit (Thermo Fisher Scientific GmbH, Dreieich, Germany). Real-time PCR was performed using the Taqman Gene Expression master mix (Thermo Fisher Scientific GmbH, Dreieich, Germany) or a mirVana™ qRT-PCR miRNA Detection Kit (Ambion, U.S.A.). Primers for miRNAs and internal control U6 were from Thermo Fisher Scientific GmbH (Dreieich, Germany), and primers for RRM2 and internal control GAPDH were designed and synthesized by Shenggong Company (Shanghai).

### Western blot analysis

Whole-cell protein extracts were prepared using RIPA lysis buffer (50  mM Tris, pH  7.4; 150 mM NaCl; 1 mM each of NaF, NaVO4 and EGTA; 1% NP40; 0.25% sodium deoxycholate; 0.2 mM phenylmethylsulphonyl fluoride; 1 μg/ml each of antipain, aprotinin and chymostatin; 0.1 μg/ml leupeptin; 4.0  μg/ml pepstatin) and detected by Western blot analysis as described [16]. Antibodies used were mouse monoclonal to RRM2 (ab57653, Abcam, Cambridge, U.K.), mouse monoclonal to caspase-3 (ab13585, Abcam, Cambridge, U.K.), mouse monoclonal to β-Actin (Sigma-Aldrich, St. Louis, MO, U.S.A.) and HRP-conjugated secondary antibody from Santa Cruz Biotechnology (Santa Cruz, CA, U.S.A.).

### Lipotransfection

MiR mimic or inhibitor or siRNA were transfected to cells with HiPerFEct Transfection Reagent (Qiagen, Hilden, Germany), according to instructions of the manufacturer. For Mock transfection, only transfection reagent was used.

siRNA-RRM2: 5′-GAUUUAGCCAAGAAGUUCAGA-3′

siRNA-Control: 5′-UAGCGACUAAACACAUCAAUU-3′

miR-20a-5p mimic: 5′-UAAAGUGCUUAUAGUGCAGGUAG-3′(MCH01529, abmgood), miRNA Mimic Negative Control (MCH00000, abmgood), miR-20a-5p inhibitor (MIH01529, abmgood) and miRNA Inhibitor Negative Control (MIH00000, abmgood) were purchased from Sigma.

### Dual-luciferase reporter assay

The putative miR-20a-5p binding site in the 3′-UTR of target gene RRM2 (wt or mut, Figure 3A) were cloned into psi-CHECK (Promega) vector downstream of firefly luciferase 3′ UTR as a primary luciferase signal with rellina luciferase as the normalization signal and termed as psiRRM2-wt and psiRRM2-mut. The psi-CHECK vector itself provided renilla luciferase signal as normalization to compensate the differences between transfection and harvested efficiencies. Transfection into HEK293 cells was performed using Lipofectamine 2000 (Invitrogen). Both Renilla and firefly luciferase activities were measured 24 h after transfection with the Dual-Luciferase Reporter Assay System (Promega, Mannheim, Germany) using a luminometer (Molecular Devices, U.S.A.). The relative Renilla luciferase activities were analyzed according to the instructions of the manufacturer (Promega, Mannheim, Germany).

### Statistical evaluation

Data obtained with established cell lines are presented as the means ± SD from at least three separate experiments, each performed in triplicate. The significance of the data was analyzed using the Student’s *t*-test for parametric data and the Mann–Whitney test with Bonferroni corrections for nonparametric data. Receiver operating characteristic (ROC) curves and the area under the ROC curve (AUC) were used to assess the feasibility of using plasma miRNA concentration as predict tool for gemcitabine resistance of pancreatic cancer. Survival curves were computed using the Kaplan–Meier method, and differences between survival curves were compared using the log-rank test. *P*<0.05 was considered statistically significant (**P<0.01, *P<0.05).

## Results

### RRM2 mediates gemcitabine resistance in pancreatic cancer cells

**Figure 1:**
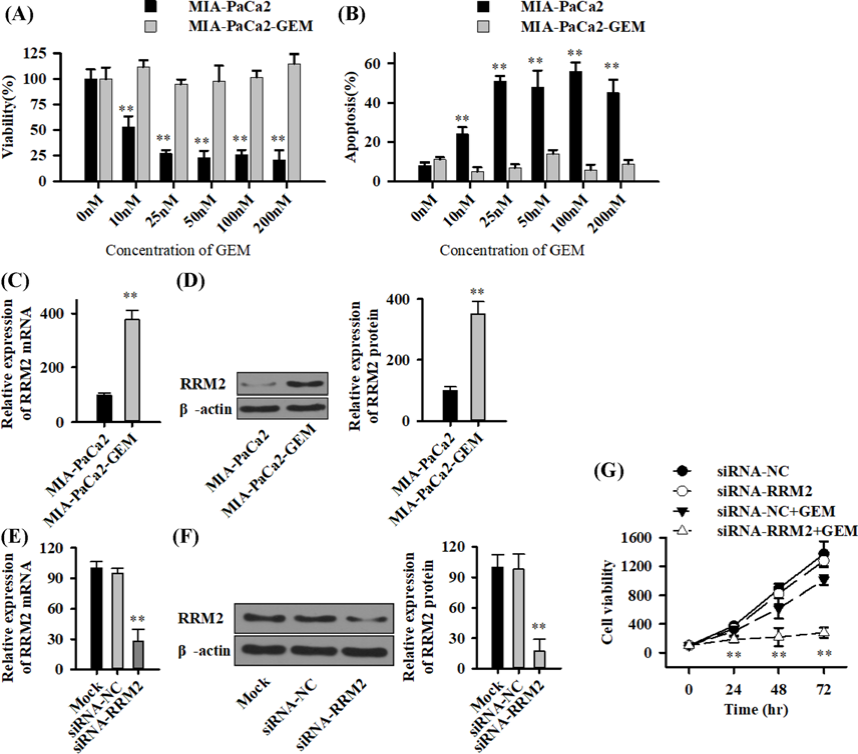
Long-time gemcitabine treatment induces resistance and RRM2 increases in pancreatic cancer cells (**A**) MIA-PaCa2 cells and the derived long-time gemcitabine-treated subclone MIA-PaCa2-GEM were untreated (0 nM) or treated with gemcitabine in concentrations from 10 to 200 nM as indicated. The viability was measured by MTT assay 72 h after treatment. (**B**) cells were treated as described above followed by staining with Annexin V-FITC and PI 72 h later and evaluation by FACS-analysis. The percentage of FITC-positive cells is shown as ‘Apoptosis (%)’. (**C** and **D**) Total RNA and cytoplasmic proteins were extracted from MIA-PaCa2 and MIA-PaCa2-GEM cells, and the expression of RRM2 was analyzed by qRT-PCR (C) and Western blot (D). GAPDH and β-actin were used as controls. (**E** and **F**) MIA-PaCa2-GEM cells were treated with 100 nM specific or negative control siRNA in the presence or absence of gemcitabine (100 nM). Cells were harvested 72 h later. The expression of RRM2 was analyzed by qRT-PCR (E) and Western blot (F). (**G**) The viability was measured by MTT assay when treated by gemcitabine (100 nM) for 24, 48 and 72 h. Each experiment was performed three times; **P<0.01.

To determine the degree of resistance of the MIA-PaCa2-derived gemcitabine-resistant subclone MIA-PaCa2-GEM, the parental and derived cells were treated with different concentrations of gemcitabine. The cell viability and apoptosis were determined 72 h later by MTT assay and staining with FITC-conjugated Annexin V/PI followed by FACS analysis. Gemcitabine inhibited the viability of parental MIA-PaCa2 cells, but MIA-PaCa2-GEM cells were totally resistant, even with the highest gemcitabine concentration of 200 nM (Figure 1A). Correspondingly, the percentage of apoptosis increased strongly in MIA-PaCa2 cells after gemcitabine treatment, whereas MIA-PaCa2-GEM cells were totally resistant (Figure 1B). In addition, RRM2 mRNA (Figure 1C) and protein (Figure 1D) were both identified as up-regulated in MIA-PaCa2-GEM cells. To further elucidate these results, specific siRNA oligonucleotides for inhibition of RRM2 (si-RRM2) were lipofected into MIA-PaCa2-GEM cells. SiRNA-NC and Mock were used as control. Seventy-two hours after transfection, qRT-PCR and Western blot demonstrated a significant down-regulation of RRM2 (Figure 1E and F). The combined treatment of MIA-PaCa2-GEM cells with siRRM2 and gemcitabine blocked gemcitabine resistance and dramatically reduced the cell viability 48 and 72 h after treatment, compared with siRNA-NC (Figure 1G).

### RRM2-related miRNAs expression and miR-20a-5p directly target RRM2 3′-UTR

**Figure 2:**
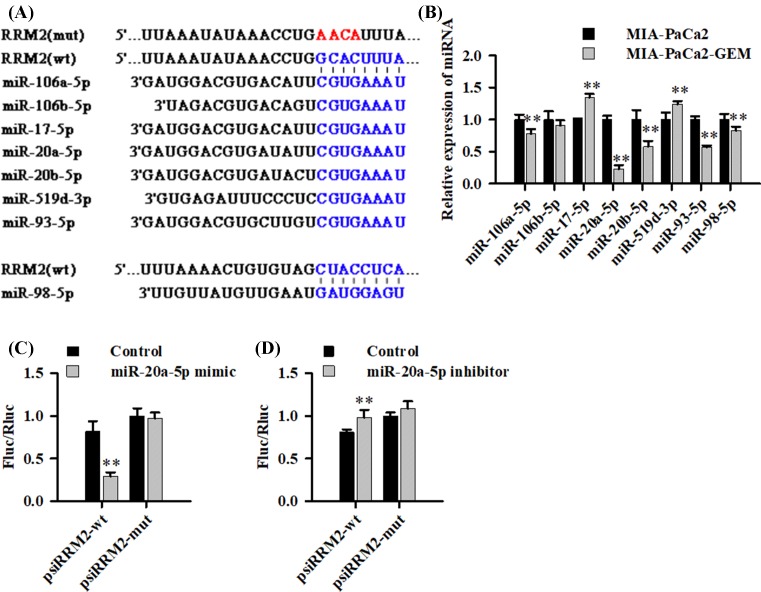
The expression of RRM2-related miRNAs and miR-20a-5p directly targets RRM2 3′UTR (**A**) Putative binding sites of miRNAs in the RRM2 3′UTR identified using the databases are shown. (**B**) qRT-PCR analysis of the relative expression of miRNAs in parental or gemcitabine-resistant cells. U6 was used as internal control, and miRNAs expression in daughter cells was normalized to parental cells. (**C** and **D**) The indicated wt- or mut-MMR2 luciferase vectors and miR-20a-5p mimic Control/ mimic (C) or miR-20a-5p inhibitor Control/inhibitor (D) was co-transfected into HEK293 cells. The luciferase activity of luciferase reporter vector was then determined. All assays were performed in triplicate and repeated at least three times (mean ± SD); **P<0.01.

To detect differences in RRM2-related miRNAs expression between MIA-PaCa2 and MIA-PaCa2-GEM cells, miRNA expression profiling was performed. Of all eight miRNAs (Figure 2A) predicted by TargetscanSites, picTarSites, RNA22Sites, PITASites or miRandaSites.org algorithms, miR-20a-5p showed the most significant differential expression (decreased ∼75%, *P*<0.01) (Figure 2B). Then, we PCR-cloned the 3′-UTRs of RRM2 with a miR-20a-5p binding site and its seed-sequence-mutated version (Figure 2A) downstream to the ORF of a Renilla luciferase reporter gene and assessed the ability of miR-20A-5p to regulate luciferase expression. The results showed that the RRM2 wild-type but not mutated 3′-UTR responded to miR-20a-5p by directing ∼65% reduction of reporter gene expression in HEK293 cells (Figure 2C). Furthermore, luciferase activity was clearly affected by the co-transfection of miR-20a-5p inhibitor and wt 3′-UTR (*P*<0.01) but not mt 3′-UTR (*P*>0.05) (Figure 2D). Taken together, these findings suggests that miR-20a-5p dramatically decreases in GEM-resistant pancreatic cancer cells and binds directly to the predicted sequence of RRM2 3′-UTR.

### miR-20a-5p inhibits protein expression of RRM2 and reverses gemcitabine resistance

**Figure 3:**
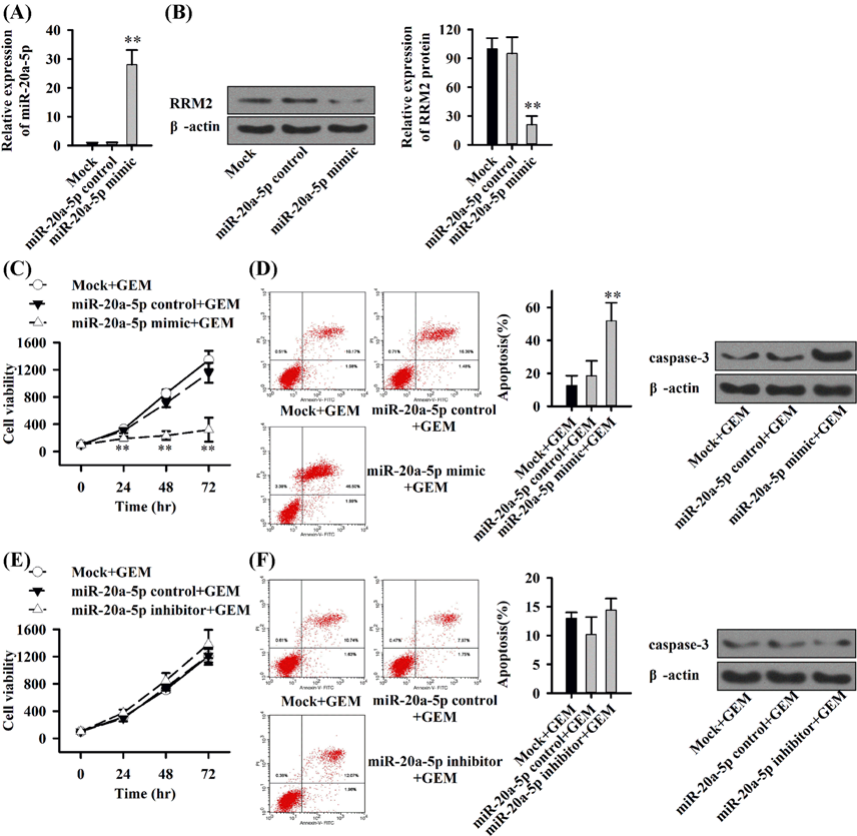
miR-20a-5p targets RRM2 to reverse gemcitabine resistance (**A**) qRT-PCR of MIA-PaCa2-GEM cells lipofected with miR-20a-5p mimic. (**B**) Western blot analysis of MIAPaCa2-GEM cells lipofected with miR-20a-5p mimic. (**C**) Time–response kinetics evaluated by MTT assay in MIA-PaCa2-GEM cells after lipotransfection with miR-20a-5p mimic or control in the presence of gemcitabine. (**D**) Apoptosis analysis in MIA-PaCa2-GEM cells staining with FITC-conjugated Annexin V/PI after lipotransfection with miR-20a-5p mimic or control in the presence of gemcitabine for 72 h; Western blot analysis of caspase-3 in MIAPaCa2-GEM cells. (**E**) Time–response kinetics evaluated by MTT assay in MIA-PaCa2-GEM cells after lipotransfection with miR-20a-5p inhibitor or control in the presence of gemcitabine. (**F**) Apoptosis analysis in MIA-PaCa2-GEM cells staining with FITC-conjugated Annexin V/PI after lipotransfection with miR-20a-5p inhibitor or control in the presence of gemcitabine for 72 h; Western blot analysis of caspase-3 in MIAPaCa2-GEM cells. Each experiment was performed three times; **P<0.01.

Based on the results above, we speculated that miR-20a-5p might block gemcitabine resistance through interfering with the expression of RRM2. The lipofection of miR-20a-5p mimic or control was used in MIA-PaCa2-GEM cells, leading to a strongly enhanced expression of miR-20a-5p in the gemcitabine-resistant cells after 48 h (Figure 3A). Simultaneously, the expression of RRM2 protein was significantly reduced (Figure 3B). Finally, we performed time-response kinetics evaluated by MTT assay (Figure 3C, 0–72 h) and apoptosis analysis (Figure 3D, after 72 h) in MIA-PaCa2-GEM cells after lipotransfection with miR-20a-5p mimic or control in the presence of gemcitabine. The combination of gemcitabine (100 nM) with mock or miR-control had no obvious effect in MIA-PaCa2-GEM cells, whereas miR-20a-5p mimic strongly reduced the viability of MIAPaCa2-GEM cells in the presence of gemcitabine (Figure 3C). Meanwhile, the combination of miR-20a-5p mimic with gemcitabine significantly enhanced the cytotoxicity in MIA-PaCa2-GEM cells (Figure 3D). But, the combination of miR-20a-5p inhibitor with gemcitabine did not affect the cell viability and cytotoxicity in MIA-PaCa2-GEM cells (Figure 3E and F). Thus, elevated miR-20a-5p had the capacity of targeting to inhibit MMR2 protein expression and reverse gemcitabine resistance in our study.

### miR-20a-5p serves as a predictor for predicting the efficacy of GEM-based chemotherapy in first-line treatment of pancreatic cancer patients

**Figure 4:**
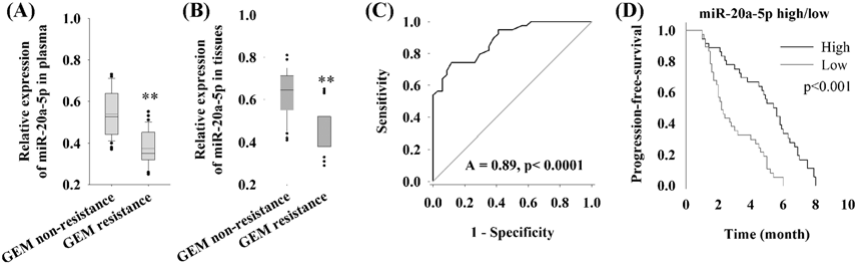
The feasibility of using plasma miR-20a-5p level as a predict tool for gemcitabine resistance of pancreatic cancer (**A**) qRT-PCR of plasma miR-20a-5p in patients with or without gemcitabine resistance. (**B**) qRT-PCR of tissue miR-20a-5p in patients with or without gemcitabine resistance. (**C**) ROC curve for plasma miR-20a-5p level as a predict tool for gemcitabine resistance in pancreatic cancer. (**D**) Kaplan–Meier analysis for PFS in pancreatic cancer patients with high and low expression level of miR-20a-5p; **P<0.01.

In the cellular experiments, we reveal that miR-20a-5p influences the sensitivity of pancreatic cell line MIA-PaCa2 to gemcitabine via regulating the expression of RRM2 protein. Then the expression of miR-20a-5p in patient’s plasma was detected, and the relationship between miR-20a-5p and the response of patient to gemcitabine-based chemotherapy was retrospectively evaluated. Results indicated that the relative expression of miR-20a-5p in gemcitabine resistant plasma of PC patients was significantly lower than in nonresistant patients (*P*<0.01) (Figure 4A). In cancer tissues, expression of miR-20a-5p in gemcitabine resistant PC patients was also significantly lower than in nonresistant patients (*P*<0.01) (Figure 4B). Furthermore, we used receiver operating characteristic (ROC) curve and the area under the ROC curve (AUC) to assess the feasibility of using plasma miR-20a-5p level as a predict tool for gemcitabine resistance in pancreatic cancer. ROC curve showed that miR-20a-5p abundant level might predict gemcitabine resistance with an AUC of 89% (*P*<0.0001) (Figure 4C). To elucidate the relationship between miR-20a-5p expression and gemcitabine-based chemotherapy, we used Kaplan–Meier analysis for PFS in pancreatic cancer patients with high and low expression level of miR-20a-5p. There were no significant differences between patients with high or low level of miR-20-5p in clinicopathological factors as described in ‘Methods’ section. The PFS of patients with high and low expression levels miR-20a-5p was 2.8 and 4.5 months (*P*<0.001), respectively (Figure 4D).

## Discussion and conclusion

At present, pancreatic cancer remains a highly malignant tumor and the prognosis is extremely poor. Gemcitabine-based chemotherapy has formed the core of the multimodal therapy and improved the prognosis of patients with pancreatic cancer [17], but its effect is modest because of high drug resistance. Screening of tissue samples for several miRNAs revealed convincing evidence that a large number of miRNAs are dysregulated in drug resistant cancer cell lines [18]. We explored the role of miR-20a-5p, one potential miRNA targeted the activity subunit of ribonucleotide reductase—RRM2, in pancreatic cancer-related gemcitabine resistance in the present study.

The drug resistance gene, RRM2, reduces the pharmacological activity of gemcitabine by affecting its metabolism in pancreatic cancer cells [19]. Previous evidence suggest that RRM2 could predict the prognosis of patients treated with gemcitabine, as relatively high expression of this gene in pancreatic cancer is associated with a poor prognosis, and patients who do not benefit from gemcitabine treatment tend to have elevated RRM2 expression [20]. In addition, down-regulation of RRM2 in pancreatic cancer cells could increase their chemosensitivity to gemcitabine [21]. We observed that mRNA and protein of RRM2 both up-regulated in established gemcitabine-resistant pancreatic cancer cells, which was consistent with previous studies [19,20]. Moreover, reduction of RRM2 expression by siRNA restored sensitivity of gemcitabine-resistant cells to this drug. These results showed that RRM2 plays an essential role in mediating gemcitabine resistance in pancreatic cancer line MIA-PaCa2, further proved the positive role of RRM2 in acquired gemcitabine resistance.

miRNAs bind to the 3′-UTR of their target mRNAs, leading to degradation of mRNA or inhibiting mRNA translation. Due to the function of miRNAs on gene expression, increasing interests in the association between miRNA expression in tumors and chemosensitivity have emerged. Our study indicated that there were several miRNAs potentially targeting RRM2 as Targetscan.org et al. predicted. One of these miRNAS, miR-20a-5p, abnormally and dramatically decreased in gemcitabine-resistant cells. Next, we found miR-20a-5p directly targeted RRM2 and reduced the expression of RRM2 protein, thus reversed the drug resistance of MIA-PaCa2-GEM cells. And indeed, different miRNAs have been found to predict sensitivity to anticancer treatment, such as miR-30c, miR-130a and miR-335 that are down-regulated in various chemoresistant cell lines [22], Let-7g and miR-181b that are strongly associated with response to 5-fluorouracil-based antimetabolite S-1 [23]. In addition, several miRNAs were shown to influence sensitivity to chemotherapy. For example, inhibition of miR-21 and miR-200b increased sensitivity to gemcitabine in cholangiocarcinoma cell lines [24]. Xia et al. [25] reported that miR-20a was down-regulated more than 2-fold in a multidrug-resistant gastric cancer cell line. Here, our study suggested that miR-20a-5p was reduced in drug-resistant pancreatic cancer cells, and it could influence gemcitabine sensitivity through negative regulating RRM2.

Therefore, evaluating the expression of miR-20a-5p in clinical samples turns to be crucial in predicting the drug-resistance, as it has been proved that RRM2 leads to less benefit from gemcitabine treatment in pancreatic cancer patients who have elevated RRM2 expression [20]. The present study revealed a significant association between miR-20a-5p expression and the clinical response to gemcitabine-based chemotherapy in pancreatic cancer patients. Patients who highly expressed miR-20a-5p benefited from gemcitabine therapy compared with low miR-20a-5p patients. And expression level of miR-20a-5p could be a newly independent predictor of the clinical response to gemcitabine in pancreatic cancer patients. Taken together, miR-20a-5p served as a predictor of gemcitabine-based chemotherapy efficacy in pancreatic cancer patients.

In conclusion, we demonstrate in the present study that RRM2 inhibits the anticancer effect of gemcitabine in pancreatic cancer cells and that miR-20a-5p negatively regulates this effect. The response to gemcitabine in Mia-PaCa2-GEM cells is controlled by genetic manipulation of miR-20a-5p and RRM2. In addition, clinical data reveal that patients who highly express miR-20a-5p benefit from gemcitabine-based chemotherapy with regard to PFS. Considered together, the results suggest that miR-20a-5p mediated RRM2-related gemcitabine resistance, and miR-20a-5p may serve as a predictor for predicting the efficacy of gemcitabine-based chemotherapy in first-line treatment of pancreatic cancer patients.

## Abbreviations

AUC: area under the ROC curve
PC: pancreatic cancer
PFS: progression-free-survival
ROC: receiver operating characteristic
RRM2: ribonucleotide reductase subunit M2
